# Improving Marine Concrete Performance Based on Multiple Criteria Using Early Portland Cement and Chemical Superplasticizer Admixture

**DOI:** 10.3390/ma14174903

**Published:** 2021-08-28

**Authors:** Taegyu Lee, Jaehyun Lee, Jaewook Jeong, Jaemin Jeong

**Affiliations:** 1 Department of Fire and Disaster Prevention, Semyung University, 65, Semyeong-ro, Jecheon-si 27136, Korea; ltg777@semyung.ac.kr; 2 Department of Safety Engineering, Seoul National University of Science and Technology, 232 Gongneung-ro, Nowon-gu, Seoul 01811, Korea; archi0528@seoultech.ac.kr (J.L.); ss96011@seoultech.ac.kr (J.J.)

**Keywords:** marine environment, chloride resistance, early Portland cement, early strength, ground granulated blast-furnace slag, time for removal of forms, life cycle CO_2_

## Abstract

This study sought to examine the performance design of concrete mix proportions to ensure chloride resistance and early strength with respect to C35 (35 MPa), which is the minimum compressive strength class of concrete used in a marine environment. For the proposed concrete mixture, C24 (24 MPa) was selected and binders for concrete were manufactured using a blend of OPC (ordinary Portland cement), EPC (early Portland cement), and GGBS (ground granulated blast-furnace slag). The results of the experiment confirmed that the combined use of EPC and GGBS greatly improve the early-strength development and chloride resistance of concrete. An analysis revealed that the time for removal of forms can be reduced by 5–9 h from the aspect of early concrete strength. Moreover, in terms of construction productivity, EPC and GGBS were reduced by up to 16.39 h/cycle compared to other concretes. Regarding economic and environmental impacts, EPC and GGBS were more effective than C35 concrete. This study is significant as its findings help make it possible to examine the most economical concrete mix design in relation to strength development according to the application of EPC, GGBS, and PC-based admixtures.

## 1. Introduction

Reinforced concrete (RC) structures in a marine environment should ensure the durability of steel-reinforced concrete against chloride ion ingress in consideration of the local-area circumstances. In concrete, a passive film is formed on the steel surface to block contact with water and oxygen and thus prevent the corrosion of the reinforcement. The presence of chloride ions (Cl^−^) in the marine environment affects the corrosion of ferrous (Fe^2+^) metals [1,2,3,4]. The corrosion of the reinforcement leads to concrete cover cracking and degrading in the RC structure due to volume expansion, resulting in the deterioration of the durability of the concrete [5,6,7,8,9].

Chloride, which is a major factor in the corrosion of the reinforcement, can be incorporated into cement, aggregates, mixing water, and admixtures, etc., in the process of concrete mix design. It can also occur in a variety of environments, such as airborne chlorides, groundwater elution, and seawater [10,11].

Therefore, to limit the chloride introduced in the process of mixing concrete, the current structural design codes [12,13,14] prescribe that quality tests be carried out in the production of concrete. The limit value of the chloride content in concrete placed through the onsite quality control does not significantly affect the reinforcement corrosion until the end of the required durability period. This is not the case, however, with chlorides introduced from the external environment, or the outside. Chloride that penetrates from the outside is continuously absorbed by the surface of the concrete due to airborne chloride ingress resulting from the effects of wind near coastal areas. In addition, concrete structures built in marine conditions and those with high groundwater levels have very high chloride concentrations in the water. Therefore, there is a need to not only control the amount of chloride during the manufacturing process but also consider the chloride penetrating from the outside [6,7,15,16,17,18].

In order to secure the durability of concrete structures by controlling the chloride that penetrates from the outside, various design standards have been proposed from different countries. Correspondingly, concrete mix design considering the thickness of the concrete cover, the increase of the concrete strength, and the replacement of the functional admixtures has been recommended in most cases. The Euro code and ACI (such as ACI 201 2016, BSI 2004, EN 13670 2010, Euro 1993, etc.) suggest that the minimum strength grade of concrete in a marine environment should be C35 (characteristic value of concrete 35 MPa) or higher [1,19,20,21]. It is also stipulated that functional admixtures such as GGBS (ground granulated blast-furnace slag), fly ash, and silica fume should be mixed to improve the chloride penetration resistance properties through the watertightness of the concrete [22,23,24,25]. Due to its long-term watertightness improvement and superior chloride ion biding capacity, GGBS has been recommended for use in coastal areas [26,27,28,29].

Meanwhile, the durability design criteria for concrete structures proposed based on national standards are excellent in terms of structural stability but cannot be considered performance design criteria considering the properties of materials. Furthermore, they can lead to excessive design depending on the regional characteristics. In addition, concrete using GGBS delays early-strength development, which may result in a decline in construction productivity. Therefore, it has been recommended that mineral admixtures like GGBS should be used to replace only a portion of the entire binder [30,31,32].

When the mineral admixture is used in concrete mix proportions, the concrete strength specification is improved to secure the strength required for the removal of forms and to reduce the construction duration. In the actual construction site, there is an urgent need for a method to promote economic efficiency and to reduce the construction duration for the improvement of the construction productivity, and it is continuously required to ensure the early strength of concrete. In addition, the demand for sustainability in consideration of the durability of concrete is continuously increasing, and research results are also being reported [33,34,35].

In this regard, this study sought to examine the performance design of concrete mix proportions to secure durability and economic efficiency compared with the C35 concrete recommended and used in coastal areas. Towards this end, the concrete mix proportion table used in the local area was utilized in the experiment. Based on the total weight of the binder, a 20 wt.% GGBS content was added to secure the chloride resistance performance of concrete, and the EPC (early Portland cement) was replaced with 50 wt.% of the total binder of OPC (ordinary Portland cement) to secure the early-strength performance of concrete. To secure the stable strength of concrete, the unit water content was reduced with the use of polycarboxylic superplasticizers to decrease the water/binder (W/B).

Based on the evaluation of the compressive strength, chloride penetration depth, chloride ion diffusion coefficient, and pore structures, the improvement of the chloride resistance of concrete was examined. In addition, productivity analysis was conducted to identify productivity improvement due to the reduction of the time for the removal of forms. Additionally, the economic and environmental impacts of concrete were analyzed.

## 2. Experiment Procedure

### 2.1. Materials

Table 1 shows the materials properties in this study. As binders for concrete used in this study, OPC, EPC, and GGBS were used. Table 2 shows the chemical compositions of the used binders. The most dominant ingredients of OPC, EPC, and GGBS are CaO, SiO_2_, and Al_2_O_3_, and the SiO_2_, Al_2_O_3_, and MgO contents of GGBS are larger than those of OPC and EPC.

**Figure 1:**
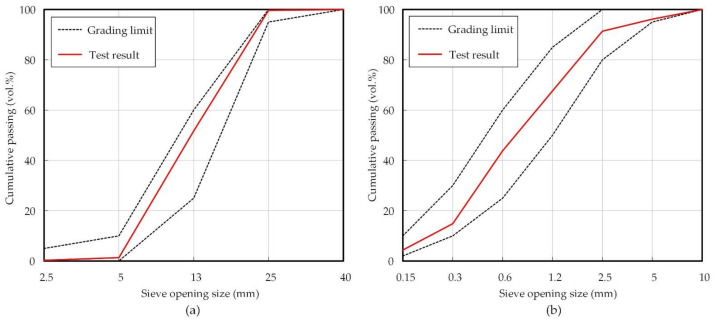
Gradation sieve curves of aggregates used: (**a**) coarse aggregates and (**b**) fine aggregates.

The mixture of washed sea sand and crushed sand was used for the fine aggregate, and a 25 mm crushed granitic aggregate was used as the coarse aggregate. Figure 1 shows the gradation sieve analysis curves of the aggregates used. The experiment was conducted after confirming that the aggregate used satisfies a range of the standard particle size distribution curve. The naphthalene group and polycarboxylic acid group were used as chemical admixtures to secure the sufficient workability of concrete.

### 2.2. Experiment Plan and Mix Proportions

**Figure 2:**
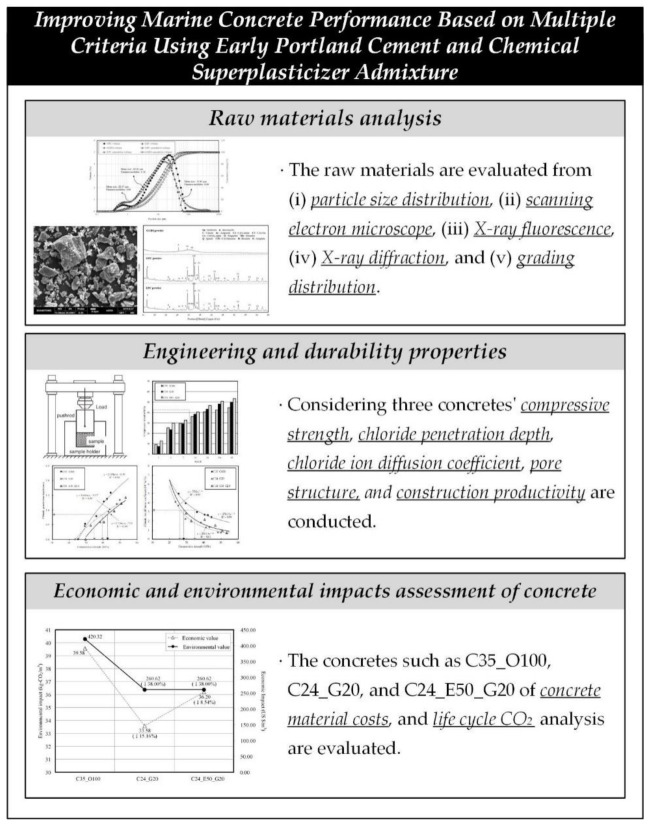
Research process.

Figure 2 shows the research process that was employed in this study, which included the following three steps: raw material analysis, engineering and durability properties, and economic and environmental impact assessment of concrete. A detailed explanation of this is as follows.

Table 3 shows the experiment plan of this study. For the analysis of concrete, raw materials were analyzed, and the physical and chemical properties of concrete were examined based on the analysis. For the analysis of raw materials, the particle size distribution, grading distribution, and the results of X-ray fluorescence, scanning electron microscopy, and X-ray diffraction were examined as evaluation items.

The compressive strength of the concrete that was used in this study was set to C35 (characteristic value of concrete 35 MPa), which is the minimum strength class for exposure conditions in areas vulnerable to chloride attack recommended by the Euro code [1,19]. For C35_O100, reference was made to the mix proportion table utilized in the actual production by a ready-mix concrete company in a local area, and 100% cement was used. In addition, a comparison experiment was conducted with reference to the mix proportion used as the mix ratio for C24 (characteristic value of concrete 24 MPa) concrete. For C24_G20, GGBS was replaced at 20% of the total binder, which is a range that does not significantly reduce compressive strength while ensuring chloride resistance. For C24_E50_G20, 50% of the EPC was replaced to increase the early strength of concrete due to the replacement of GGBS. In addition, polycarboxylic superplasticizer was used in combination to improve the workability and strength of concrete.

The evaluation items included the compressive strength, chloride penetration depth, chloride ion diffusion coefficient, and pore structure.

Table 4 shows the concrete mix proportions in this study. For the mix proportions that were used in this study, the mix table for concrete produced at the ready-mix concrete plant was used. In the case of C35_O100, the unit water content was 185 kg/m^3^, and the amount of OPC used was 444 kg/m^3^. For C24_G20 and C24_E50_G20, the unit water content was 163 kg/m^3^, and the powder content was 340 kg/m3. Based on the existing literature, the quantity of GGBS replacement was set to 20% of the total unit weight of binder considering the long-term strength development of concrete. The concrete slump was set to 180 ± 25 mm to satisfy sufficient workability considering the construction site conditions, and the air contents (%) were set to satisfy the 4.5 ± 1.5% range.

### 2.3. Experiment Method

#### 2.3.1. Binder Raw Material Analysis

Table 5 shows the test methods for the raw materials of binders. For the binder raw material analysis, the particle size distribution was measured in accordance with ASTM C204 [36], SEM was done in accordance with ASTM C1723 [37], XRF was done in accordance with ASTM C114 [38], and XRD was done in accordance with ASTM C1365 [39].

#### 2.3.2. Fresh and Hardened Properties of Concrete

To evaluate the fresh properties of concrete, a slump test was conducted in compliance with ASTM C143 [40], and the air contents were measured in accordance with ASTM C231 [41]. The evaluation of concrete compressive strengths was conducted on the Ø100 × 200 mm cylindrical specimens at set ages. For the test results, the average value of all the three specimens was obtained in accordance with ASTM C873 [42] and ASTM C39 [43].

#### 2.3.3. Durability Properties of Concrete

Table 6 shows the test methods for the durability properties of concrete. The specimen was coated with epoxy resins except for the top surface so that the chloride could penetrate only from one side. The specimen was taken out of the solution at the established ages of 3, 7, 14, 28, 56, and 91 days and was split. The surface was then sprayed with 0.1 N AgNO_3_ solution for measuring the discoloration depth.

The chloride diffusion coefficient was evaluated in accordance with the Nordic standard NT BUILD 492 [44]. For the fabrication of a specimen used for the chloride diffusion test, part of the cylindrical specimen was cut into Ø100 × 200-mm-thick disks. The cut specimen was exposed to a 0.3 N NaOH solution (NaOH 12 g in 1 L of water) at the positive electrode, and to a 10% NaCl solution (NaCl 100 g + distilled water 900 g) at the negative electrode, to evaluate the current value when a 30 V voltage was applied. The test was conducted by selecting the applied voltage level and the duration of the test based on the evaluated current value. The chloride penetration depth was determined using AgNO_3_ solution, and the chloride diffusion coefficient was calculated using Equations (1)–(3).

(1) $$D_{nssm}=\frac{RT}{zFE}\cdot \frac{X_{d}-\alpha \sqrt{X_{d}}}{t}$$

(2) $$E=\frac{U-2}{L}$$

(3) $$\alpha =2\sqrt{\frac{RT}{zfe}}\cdot erf^{-1}\left(1-\frac{2C_{d}}{C_{0}}\right)$$

where *D_nssm_* is the non-steady-state migration coefficient (m^2^/s); *R* is the gas constant, *R* = 8.314 J/(K·mol); *T* is the average value of the initial and final temperatures in the anolyte solution; *z* is the absolute value of the ion valence for chloride, *z* = 1; *F* is the Faraday constant, *F* = 9.648 × 10^4^ J/(V·mol); *L* is the thickness of the specimen, m; *U* is the absolute value of the applied voltage, *V*, *K*; *X_d_* is the average value of the penetration depths, m; *t* is the test duration, seconds; *erf*^−1^ is the inverse of the error function; *C*_0_ is the chloride concentration in the catholyte solution, *C*_0_ ≈ 2 N; and *C_d_* is the chloride concentration at which the color changes, *C_d_* ≈ 0.07 N for OPC concrete.

The test method used for the chloride penetration depth was in accordance with NT Build 443 [45]. The concrete specimen was fabricated with a 100 × 100 × 400 mm size and was immersed in a 2.8 M NaCl brine solution until the planned age. There were three concrete specimens used in the experiment, and the average value was derived by measuring three places except for the poured upper surface.

The porosity of the inner concrete was examined to evaluate its influence on the compressive strength and air permeability according to the changes in the porosity of concrete at a given age. The test on the porosity of concrete was performed in accordance with ASTM D 4404 [46]. In general, mercury intrusion porosimetry (MIP), which is a technique used for porosity evaluation, is based on the capillary phenomenon, in which a liquid penetrates the micropores. Pressure from the outside is required for non-wetting liquids such as mercury to penetrate the pores. The smaller the pore is, the greater the pressure needed to penetrate it. Through the correlation between the pressure and the pore, the measurement result can be represented as a function of the cumulative penetration volume or the volume of mercury depending on the pressure (or the pore size).

#### 2.3.4. Assessment of the Construction Productivity on Concrete

Based on the elapsed time of concrete (C35_O100, C24_G20, and C24_E50_G20), the construction productivity was analyzed using discrete event simulation. The results showed how much elapsed time affects the construction productivity through the Discrete Event Simulation (DES). Before analyzing the construction productivity, it was important to select the target project [47]. Multi-family housing is one of the most common construction projects. Moreover, several previous researches explored the productivity of structure works in a multi-family housing project [48,49]. Therefore, structure works in a multi-family housing project was selected as a case study for the following reasons. In terms of the construction project, the multi-family housing project accounted for 26% of total construction revenue, which was very large in proportion to other construction projects in South Korea [50]. In terms of the construction works, the structure works which included form installation work, rebar arrangement work, and concrete pouring work, took up to 50% of the total construction project period and 45% of the total construction cost in a multi-family housing project. Therefore, the structure works should be focused on to improve construction productivity [49].

The data were the number of workers and working hours which were established to analyze the construction productivity using DES. The concrete curing hour was considered by elapsed time on concretes. The data were obtained from a practical expert who worked as a general contractor in the multi-family housing project. The collected data was presented in the Supplementary Materials (See Tables S1 and S2). The construction productivity was calculated by using Stroboscope, which was one of the construction productivity simulations as DES. Stroboscope could take into consideration the diversity of resource characteristics relative to other DESs [51]. In the multi-family housing project, the structure works composed start and finish on each floor. Therefore, one cycle indicated the frequency of the structure works on one floor from conducting marking to checking the strength. In this study, the data were insufficient because only one case study had been collected. Thus, this study used a triangular distribution, which was not affected by the number of data and could involve easy and accurate collection of data [49]. The DES was therefore set with 1000 cycles. The DES diagram can be seen in the Supplementary Materials (See Figure S1).

#### 2.3.5. Assessment of Economic and Environmental Impacts on Concrete

For economic and environmental impact assessments, the sum of material costs was obtained and life cycle CO_2_ analysis of concrete (C35_O100, C24_G20, and C24_E50_G20) was conducted. The following two assumptions were established to obtain the results: (i) the analysis approach; and (ii) the significant cost of ownership [47,52,53,54].

1. Analysis approach: In terms of the economic impact, the data of the material unit cost (USD/kg) were obtained from a concrete product manufacturing company in South Korea. In terms of the environmental impact, the data of the material unit CO_2_ emissions (CO_2_-kg) were obtained from the life cycle inventory data in South Korea. The material unit cost (USD/kg) and unit CO_2_ emissions (CO_2_-kg) are presented in Table 7 [55,56,57,58,59,60].
2. Significant cost of ownership: In terms of the economic impact, to present the economic impacts, the cost of the concrete materials is displayed. The cost of concrete materials is calculated by multiplying material unit weight (refer to Table 4) with material unit cost (refer to Table 7) using Equation (4).
  (4) $$CC=\sum material\;unit\;weight\;\left(kg/m^{3}\right)\times material\;unit\;cost\;\left(USD/kg\right)$$
  where *CC* is the cost of the concrete material (USD/m^3^).

In terms of the environmental impact, the LCCO_2_ was calculated using Equation (5).

(5) $$CI_{i}=\sum CI_{i}=\sum \left(Load_{i}\times eqv_{i}\right)$$

where *CI_i_* is the characterized impact of category (*i*); *CI_i_* is the characterized impact of list item (*i*); *Load_i_* is the environmental load of list item (*i*); and *eqv_i_* is the characterization coefficient value of list item (*i*).

In terms of the economic and environmental impacts, the costs and CO_2_ content of concrete (C35_O100, C24_G20, and C24_E50_G20) were considered from the life cycle perspective.

## 3. Experiment Results

### 3.1. Results of the Binder Raw Material Analysis

**Figure 3:**
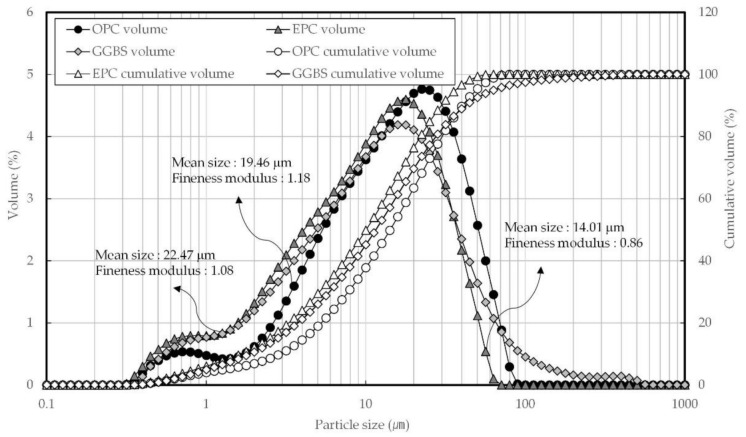
The particle size distribution of OPC (ordinary Portland cement), EPC (early Portland cement), and GGBS (ground granulated blast-furnace slag).

**Figure 4:**
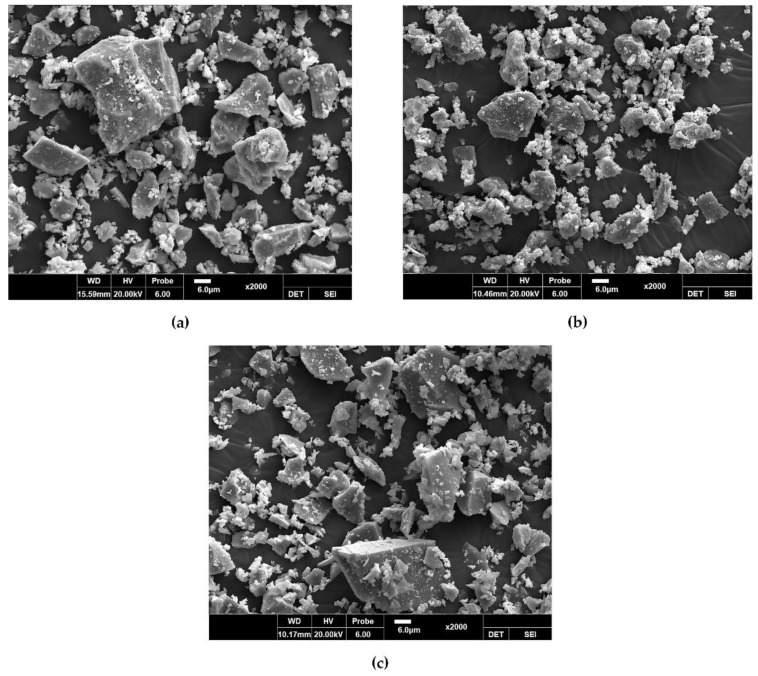
Scanning electron microscope micrograph: (**a**) OPC; (**b**) EPC; (**c**) GGBS.

Figure 3 shows the particle size distributions of OPC, EPC, and GGBS. The mean size and fineness modulus were determined to be 19.46 µm and 1.18, respectively, for OPC; 14.01 µm and 10.86 for EPC; and 22.47 µm and 1.08 for GGBS. The number of fine particles was highest in EPC, followed by GGBS and then OPC, while coarse particles larger than 10 µm were most common in OPC. Figure 4 shows the SEM micrograph. OPC was observed to have had irregular polyhedral particles larger than 10 µm and amorphous crystals smaller than 10 µm. The GGBS and EPC particles were found to have had irregular geometries but generally showed fine crystalline phases.

**Figure 5:**
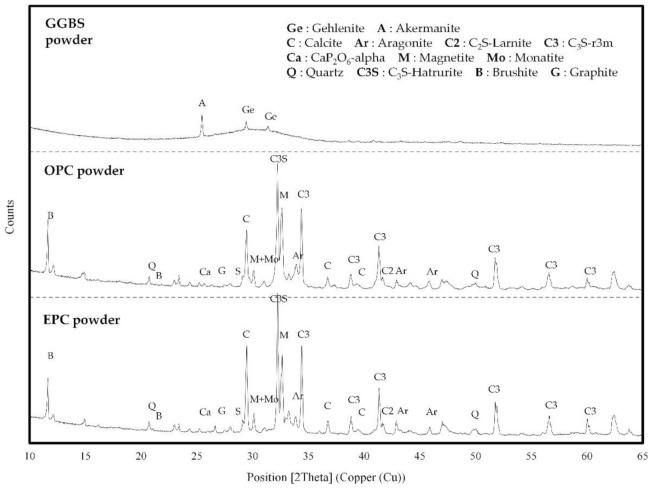
X-ray diffraction patterns of GGBS, OPC, and EPC.

Figure 5 shows the XRD patterns of OPC, EPC, and GGBS. Both OPC and EPC are mainly made up of minerals like C_3_S (3CaO∙SiO_2_), C_2_S (2CaO∙SiO_2_), C_3_A (3CaO∙Al_2_O_3_), and C_4_AF (4CaO∙Al_2_O_3_∙Fe_2_O_3_). It was found that a higher C_3_S crystalline phase was formed in EPC than in OPC, which suggests that it is more effective during the early-strength development. GGBS is composed mostly of gehlenite and akermanite.

**Figure 6:**
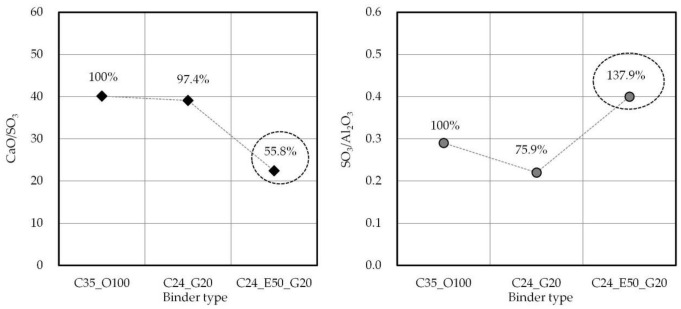
CaO/SO_3_ and SO_3_/Al_2_O_3_ ratios in binder type.

Figure 6 shows the CaO/SO_3_ and SO_3_/Al_2_O_3_ by binder type. The CaO/SO_3_ was 97.4% for C24_G20 and 55.8% for C24_E50_G20 compared with C35_O100. In addition, the SO_3_/Al_2_O_3_ was 75.9% for C24_G20 and 137.9% for C24_E50_G20 compared to C35_O10. Therefore, it was confirmed that EPC with a relatively higher SO_3_ content is more advantageous for early-strength development. The results of the research conducted by Lee et al. reported that an SO_3_ content less than 5% is advantageous for early-strength development under similar CaO content conditions [61,62,63,64]. Therefore, it is expected that in the case of C24_E50_G20, EPC incorporation can contribute to the early-strength development of concrete.

### 3.2. Fresh and Hardened Properties on Concrete

Table 8 shows the fresh properties of concrete. It was found that in all the concrete mix proportions, the target value (180 ± 25 mm) was satisfied immediately after the mixing and at 60 min of elapsed time. In the case of the C35_O100 mix, the slump decline was 35 mm at 60 min of elapsed time, but 15 mm was found in the other mix proportions. The air content of concrete met the target air volume of 4.5 ± 1.5% in all the mix proportions. Even after a 60-min elapsed-time change, similar results were found in all the mix proportions.

**Figure 7:**
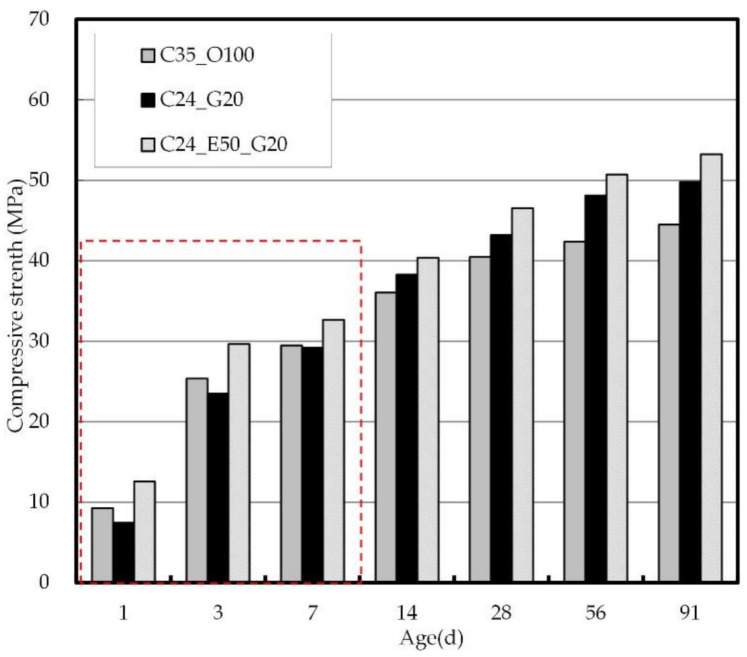
Compressive strength of concrete by age.

Figure 7 shows the compressive strength of concrete at different ages. With respect to the compressive strength of concrete by mix proportion, C24_E50_G20 exhibited the highest value, whereas C24_G20 showed the lowest value at the early ages of less than or equal to 7 days. Meanwhile, after 7 days, the strength of C24_G20 continued to increase, and the long-term strength was higher than that of C35_O100. The early strength of C24_E50_G20 was high, and the long-term strength development was also found to continue. It is known that EPC has a high C_3_S content and higher fineness, which is advantageous for the early-strength development of concrete due to the heat of hydration but is unfavorable in terms of long-term strength development [62,65].

In this study, however, the incorporation of GGBS, which is advantageous for long-term strength development, was found to make up for the shortcomings of EPC and to have a positive influence on long-term strength development. It was also found that the use of a PC-based superplasticizer could reduce the unit water content of concrete despite the low power content compared to C35_O100, thereby contributing to the development of the compressive strength. Therefore, it is expected that the use of EPC and GGBS as binders in combination with a PC-based superplasticizer will make an effective contribution to the development of the compressive strength of concrete while significantly reducing the total binder amount.

#### 3.2.1. Chloride Penetration Depth and Chloride Ion Diffusion

**Figure 8:**
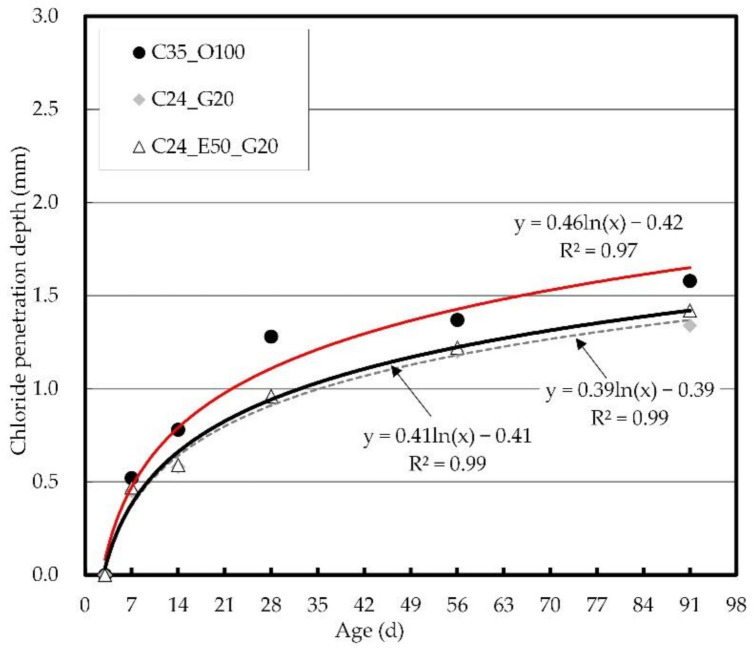
Chloride penetration depth by age.

Figure 8 shows the chloride penetration depth at different ages. The chloride penetration depth could not be measured because penetration did not occur until the early age of 3 days. It was then confirmed that after 7 days, chloride penetration of concrete began, and the penetration depth increased with age in all the mix proportions. Meanwhile, C35_O100 was found to have the largest chloride penetration depth, and the difference varied significantly depending on the age. C24_G20 and C24_E50_G20 exhibited similar chloride penetration depths.

**Figure 9:**
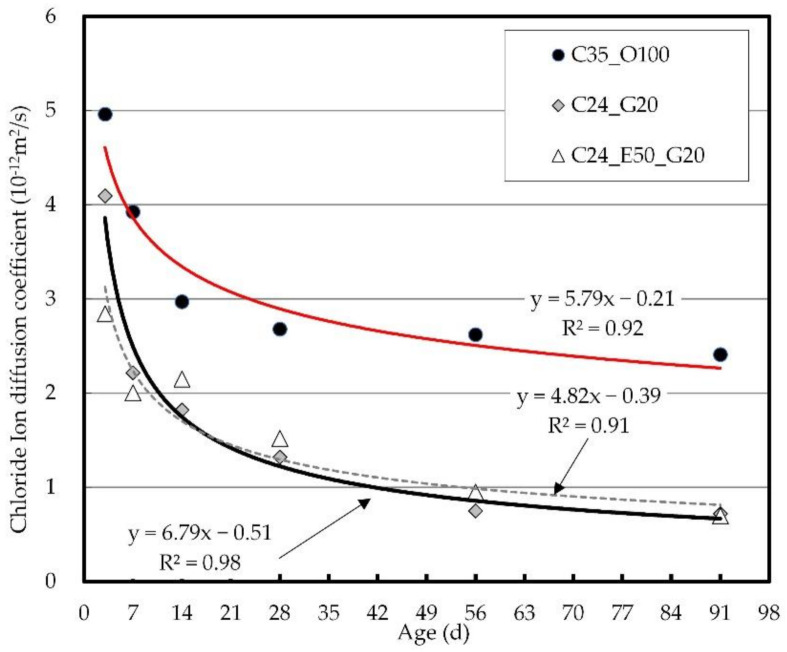
Chloride ion diffusion coefficient by age.

Figure 9 shows the chloride ion diffusion coefficient at different ages. The evaluation of the chloride ion diffusion coefficient is a criterion for reviewing long-term chloride resistance through an acceleration test. The test revealed that in all the mix proportions, the chloride diffusion coefficient continuously decreased after the early-strength development, and the chloride diffusion coefficients converged after 91 days. The C35_O100 exhibited a gradual decrease from 5 × 10^−12^ m^2^/s at the early ages to 2.4 × 10^−12^ m^2^/s at 91 days, showing a 1/2 decrease, while C24_G20 and C24_E50_G20 exhibited patterns similar to those of the chloride penetration depth results and showed a gradual decrease from 4.0 × 10^−12^ to 0.7 × 10^−12^ m^2^/s at 91 days, indicating a 1/7 decrease. It was confirmed that the value was only about 30% of the chloride diffusion coefficient compared to C35_O100.

#### 3.2.2. Porosity of Concrete

**Figure 10:**
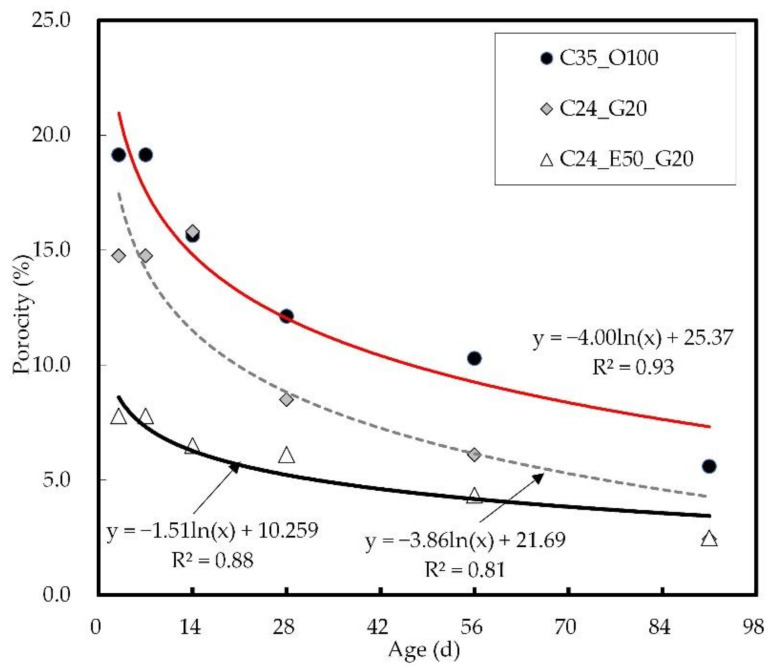
Result of internal porosity of concrete by age.

Figure 10 shows the results of the internal porosity of concrete by age. The internal porosity of concrete tended to decrease with increasing age, and this tendency was most dominant in C35_O100. The porosity of C35_O100 was about 20 vol.% at the early ages but decreased to 5.6 vol.% at 91 days. The C24_G20 exhibited 15 vol.% porosity at the early ages, but the internal porosity decreased significantly with time, showing about a 2.5 vol.% value. C24_E50_G20 showed 7.8% porosity at the early ages and then a gradual decrease to a final value of 2.5 vol.%.

**Figure 11:**
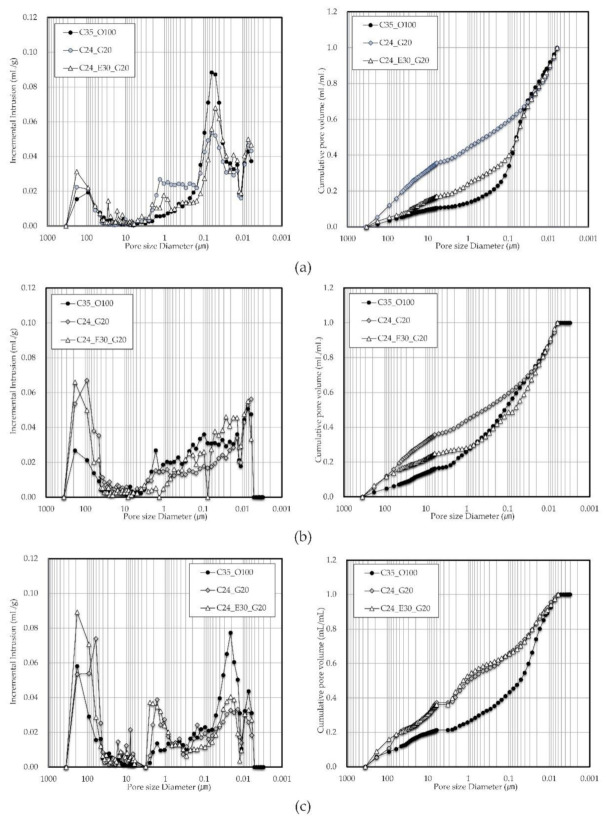
Results of pore structure by mercury intrusion porosimetry: (**a**) 7 days; (**b**) 28 days; (**c**) 91 days.

Figure 11 shows the pore structure results from mercury intrusion porosimetry. With regard to the internal porosity of each mix proportion, the pore size distributions can be divided into 100–1000 and 1–0.01 µm ranges, and the pore size ranging from 0.01 to 1 µm increased. In addition, the results of the accumulated amount of pores revealed that the 0.1–10 µm pore size range was most common in C24_G20, which showed a porosity distribution similar to that of C24_E50_G20 with increasing age. It was also confirmed that the incorporation of GGBS significantly influences the formation of pores with a 0.1–10 µm size.

## 4. Discussion

### 4.1. Effect of EPC and GGBS on the Compressive Strength and Chloride Resistance of Concrete

**Figure 12:**
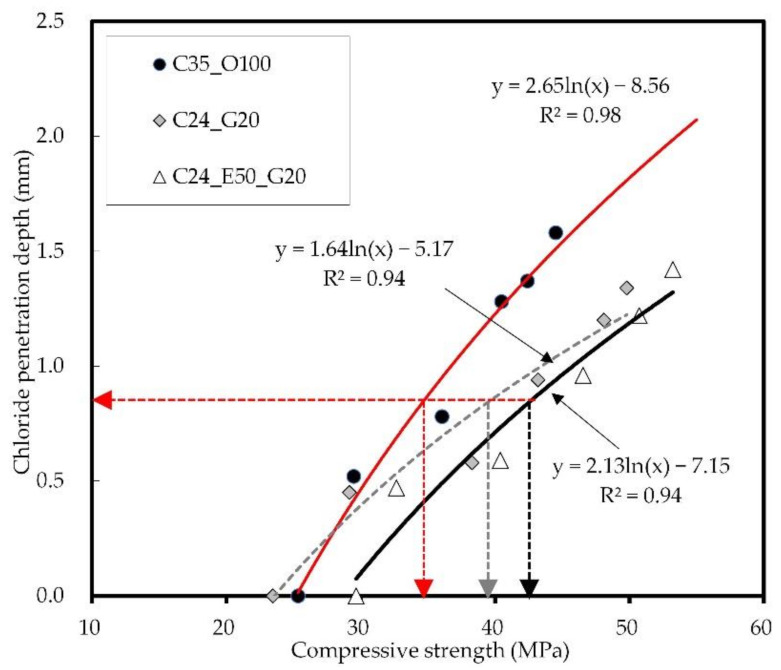
Relation between concrete strength and chloride penetration depth.

Figure 12 shows the relation between the compressive strength and chloride penetration depth of concrete. The chloride penetration depth continued to increase even after the C35 strength development stage presented in the Euro code was reached. The slope of the curve was smaller in C35_O100 than in C24_G20 and C24_E50_G20 using GGBS. It was confirmed that at the early ages, C24_E50_G20 is advantageous for chloride penetration resistance as it exhibited further progress in strength development in this study compared to C24_G20. Meanwhile, C24_E50_G20 and C24_G20 had similar strengths at the long-term age of 91 days, but GGBS was assumed to be advantageous at the later ages.

**Figure 13:**
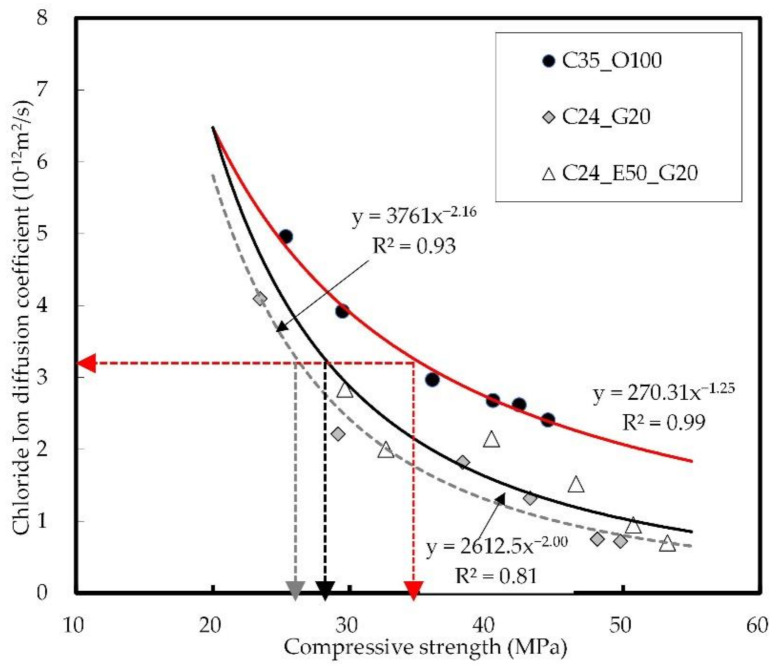
Relation between concrete strength and chloride ion diffusion coefficient.

Figure 13 shows the relation between the concrete strength and chloride ion diffusion coefficient of concrete. As mentioned earlier, it is difficult to measure the chloride penetration depth of concrete in the long term and to accurately analyze its effects due to the influence of chloride accumulation on it. In the case of the chloride ion diffusion coefficient, however, its performance can be examined through a chloride acceleration test for estimation from a long-term perspective.

When the compressive strength of C35_O100 is 35 MPa, the chloride ion diffusion coefficient is 3.2 × 10^−12^ m^2^/s. To achieve the same performance, C26 is required for C24_G20 and C28 for C24_E50_G20. It was confirmed that the sole use of GGBS ensures the greatest effectiveness in chloride resistance performance by the chloride ion diffusion coefficient. Continuous exposure to chloride at the early ages, however, leads to an increase in the concentration of surface chloride ions, which may increase the chloride diffusion coefficient. Therefore, the use of EPC needs to be considered to ensure chloride ion penetration resistance through the surface watertightness at the early ages. In this connection, it is expected that the additional incorporation of GGBS will make it possible to improve the internal watertightness of the blast-furnace slag and the chloride ion binding capacity, thus ensuring satisfactory performance [66,67].

### 4.2. Relation between the Porosity and Compressive Strength of Concrete and Chloride Resistance

**Figure 14:**
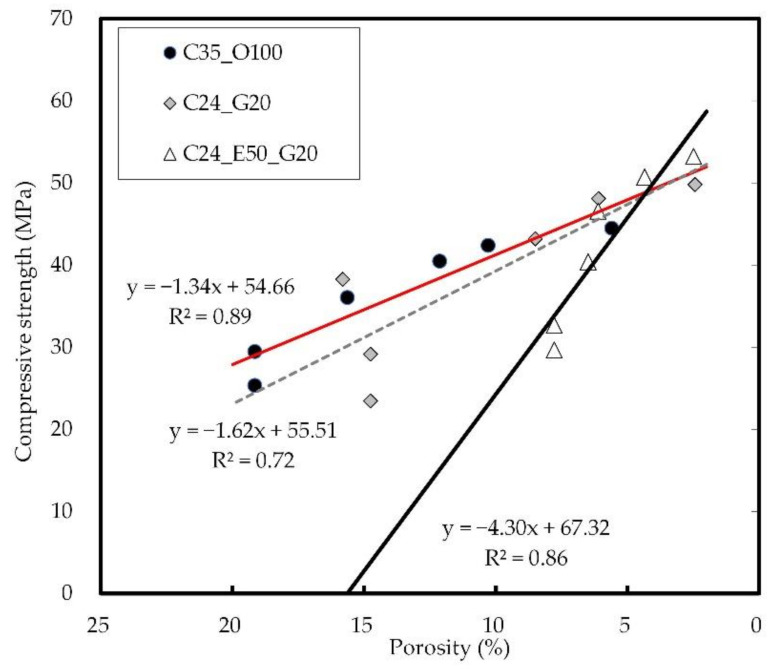
Relation between internal porosity and compressive strength on concrete.

Figure 14 shows the relation between the internal porosity and concrete strength of concrete. It was confirmed that the two generally have a linear relationship. In C35_O100 and C24_G20, the strength developed as the porosity decreased, indicating a similar pattern. For C24_E50_G20 incorporating EPC, the porosity was low due to rapid reactivity at the early ages, which is advantageous for early-strength development (see Figure 6).

**Figure 15:**
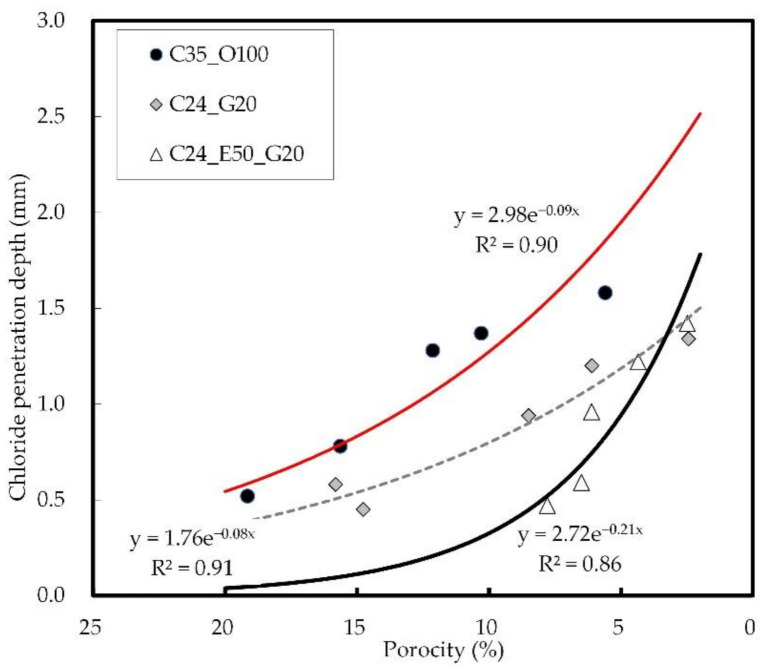
Relation between internal porosity and chloride penetration depth on concrete.

Figure 15 shows the relation between the internal porosity and the chloride penetration depth of concrete. The chloride penetration depth increased even when the porosity decreased, and C35_O100 exhibited a higher value than C24_E50_G20 and C24_G20 under the same porosity conditions. In addition, the difference between C24_G20 and C35_O100 tended to be larger as the porosity decreased, which suggests that the effect of reducing the chloride ion penetration rate of concrete becomes greater in this case. It was confirmed that C24_E50_G20 showed an increase in chloride penetration depth as the porosity decreased, but it eventually exhibited a value similar to that of C24_G20.

**Figure 16:**
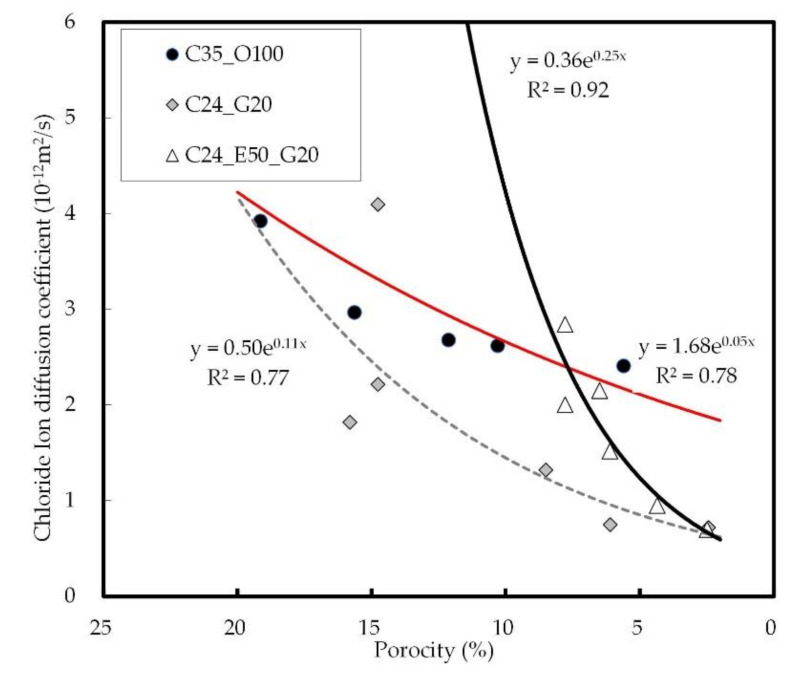
Relation between internal porosity and chloride ion diffusion coefficient on concrete.

Figure 16 shows the relation between the internal porosity and the chloride ion diffusion coefficient of concrete. The chloride ion diffusion coefficient decreased as the porosity of concrete decreased, and this tendency was identified as most pronounced in C24_E50_G20, followed by C24_G20 and then C35_O100. It was reported in a study conducted by Sakai (2019) that there is a high correlation between the internal porosity and the chloride ion diffusion coefficient of concrete [68,69]. In this study, it was confirmed that the chloride penetration is reduced by the formation of micropores due to the effects of GGBS at long-term ages when the internal porosity decreases as the initial reactivity increases with the use of the PC-based superplasticizer.

### 4.3. Analysis of the Removal of Forms

**Figure 17:**
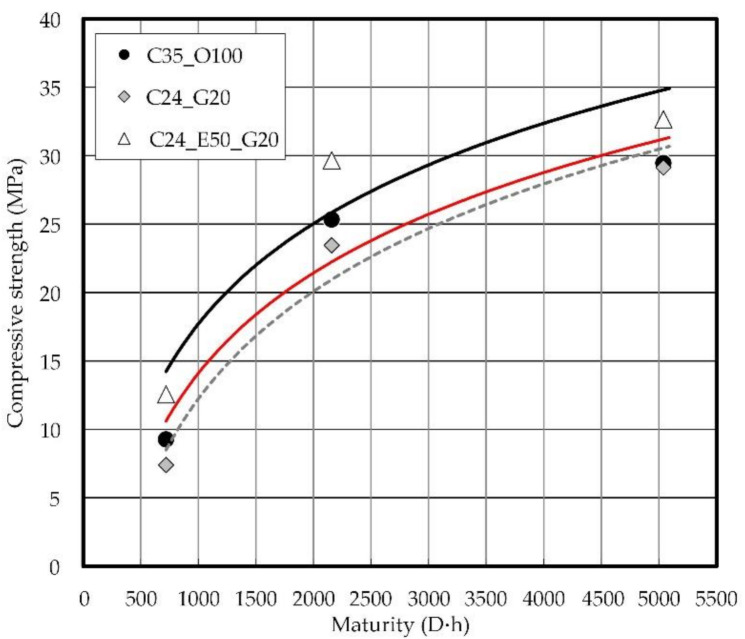
Relation between maturity and compressive strength on concrete.

This study revealed that the use of EPC and chemical superplasticizer in concrete mix proportions makes it possible to reduce the quantity of cement and the unit water content of the existing C35 concrete mix as well as to secure the early strength of concrete. Figure 17 shows the relation between the maturity and the compressive strength of concrete. The evaluation of the resistance to chloride attack confirmed that C24_G20 has slightly superior performance compared to C24_E50_G20 but is disadvantageous in terms of the early strength of concrete. If the correlation between the average curing temperature and the compressive strength of concrete is derived from the relationship between the maturity and the compressive strength, it is possible to predict the strength of the removal of forms [70]. The early-strength development of concrete has many advantages in increasing the productivity of construction sites.

The standard time for the removal of vertical forms varies by country. In the U.S. and Europe, the removal time period is defined as the elapsed time according to the curing temperature (15 °C or higher, 12 h), while in Asia (Japan and South Korea), the point in time of 5 MP of strength development is defined as the formwork removal time. Therefore, this study was conducted based on the Asian standards presenting detailed strength values.

**Figure 18:**
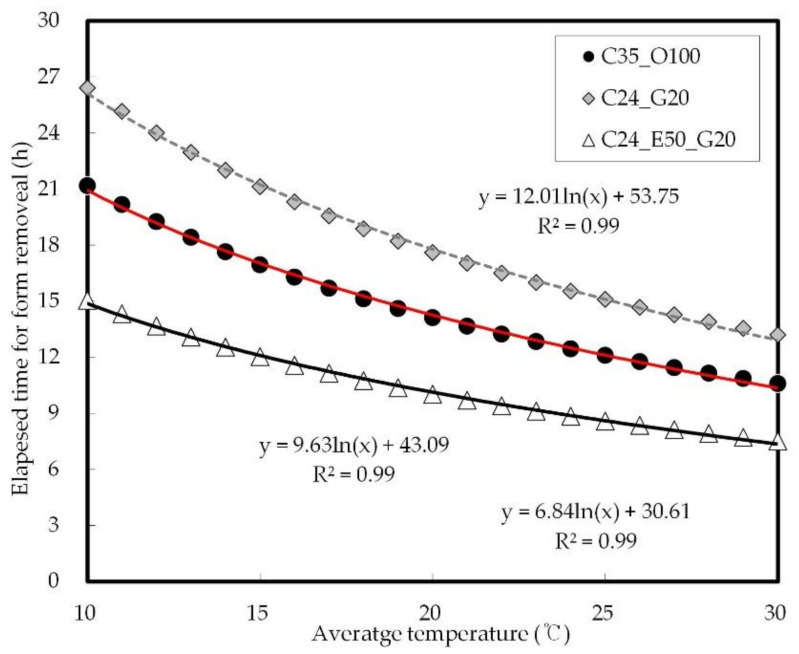
Prediction of elapsed time at a strength of 5 MPa on concrete according to curing temperature.

Figure 18 shows the prediction of the elapsed time at 5 MPa concrete strength according to the curing temperature. It was confirmed that at an average curing temperature of 15 °C, the elapsed time is 21 h for C24_G20, 17 h for C35_O100, and 12 h for C24_E50_G20. With respect to chloride resistance, similar results were obtained for C24_G20 and C24_E50_G20. C24_E50_G20, however, was found to be superior in early strength. Therefore, the mix proportions of C24_E50_G20 need to be applied for construction productivity.

### 4.4. Analysis of Construction Productivity on Concrete

**Figure 19:**
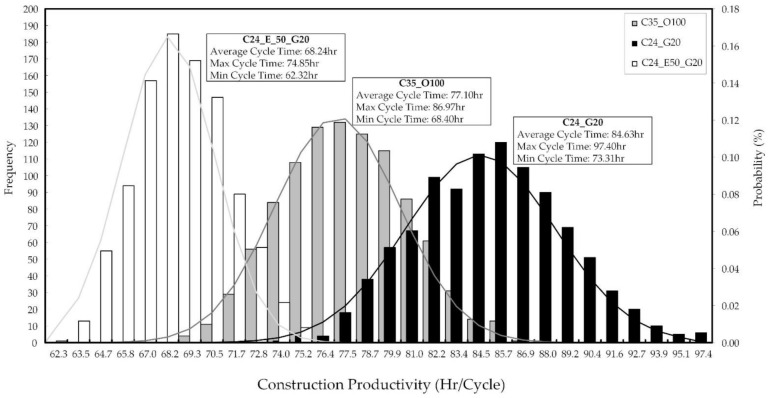
Result of the construction productivity analysis based on elapsed time.

Based on the elapsed time of concrete (C35_O100, C24_G20, C24_E50_G20), a construction productivity analysis was conducted to validate how early strength can affect construction productivity. Figure 19 shows the construction productivity result at cycle times for the typical floor in a multiplex house. Each simulation was conducted 1000 times to analyze the construction productivity on concretes.

When comparing the construction productivity of concretes, the results are as follows: C35_O100 was 77.10 h/cycle; C24_G20 was 84.63 h/cycle; and C24_E50_G20 was 68.24 h/cycle. C24_E50_G20 was superior to other concretes in terms of construction productivity. As mentioned above, the elapsed time for C24_E50_G20 was up to nine hours different from other concretes. The construction productivity, however, was up to 16.39 h different from other concretes. Because the waiting time was caused by elapsed time, the other construction works could be affected and delayed. Therefore, when C24_E50_G20 is applied to an actual construction project, it results in time-saving (see Figure 19) [71].

### 4.5. Analysis of Economic and Environmental Impacts of Concrete

**Figure 20:**
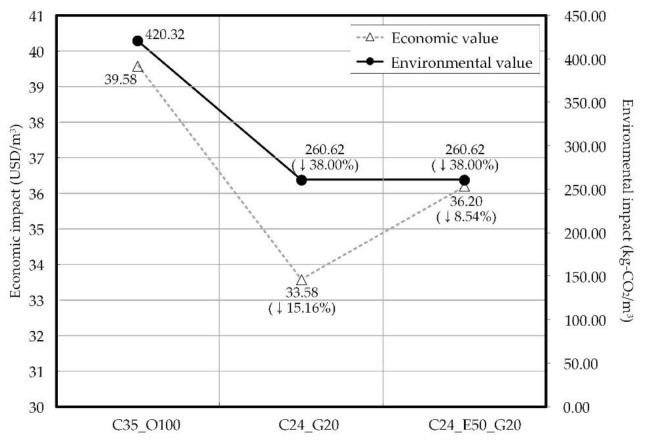
Results of environmental and economic impacts on concrete.

This study analyzed the economic and environmental impacts of C35_100, C24_G20, and C24_E50_G20 from the life cycle perspective. Figure 20 shows the economic and environmental impact results.

In terms of the economic impact, the economic analysis on concrete is as follows: C35_O100, 39.58 USD/m^3^; C24_G20, 33.58 USD/m^3^; and C24_E50_G20, 36.20 USD/m^3^. C35_O100 is more expensive than C24_G20 and C24_E50_G20 for two reasons. First, the cement cost is higher than other materials’ costs, such as GGBS, sand, and gravel (see Table 7). Second, as mentioned in the previous section, C35_O100 contains more cement than C24_G20 or C24_E50_G20. As C35_O100 is more expensive than C24_G20 and C24_E50_G20, when it is replaced with C24_G20 and C24_E50_G20, the economic impact can be reduced by up to 15.16% and 8.54%, respectively.

In terms of the environmental impact, the LCCO_2_ is as follows: C25_O100, 420.32 kg-CO_2_/m^3^; C24_G20, 260.62 kg-CO_2_/m^3^; and C24_E50_G20, 260.62 kg-CO_2_/m^3^. The LCCO_2_ of C35_O100 is higher than those of C24_G20 and C24_E50_G20. As cement emits more CO_2_ than other materials, such as water, GGBS, sand, and gravel (see Table 7), C25_O100 has more cement than other concretes. Thus, when C24_G20 or C24_E50_G20 is used instead of C35_O100 in the actual construction site, the environmental impact can be reduced by 38.00%.

## 5. Conclusions

In this study, the chloride resistance performance of concrete was evaluated, and life cycle assessment was conducted on concrete using a binder based on early Portland cement (EPC) and chemical superplasticizer admixture. The results of the analysis of the experiment results are summarized as follows.

(1) The analysis of raw materials revealed that EPC can help improve the early-strength development of concrete. In addition, it was confirmed that the use of EPC and ground granulated blast-furnace slag (GGBS) as concrete binders in combination with a PC-based superplasticizer can improve the early and long-term strengths of concrete while significantly reducing the total binder.
(2) The results of the chloride ion diffusion coefficient and chloride penetration depth showed that when EPC and GGBS are used, chloride resistance is improved by the watertightness of the surface and by the chloride ion binding phenomenon as the rapid strength development progresses at early ages. In particular, its chloride diffusion coefficient was decreased significantly by about 30% compared with C35_O100.
(3) The evaluation of the internal porosity of concrete confirmed that the mix proportions using EPC and GGBS could reduce the total pore volume and increase the watertightness of concrete, thereby improving its chloride resistance. It was also confirmed that C24_E50_G20 and C24_G20 have different degrees of porosity at early ages but exhibit almost similar degrees of porosity at long-term ages.
(4) The analysis of the effects of EPC and GGBS on the compressive strength and chloride resistance of concrete revealed that the application of a chemical superplasticizer to concrete makes it possible to reduce the cement quantity and the unit water content of the existing C35 concrete mix. Based on the study results, it is possible to secure high chloride resistance at a low strength compared to C35_100, and to reduce the time for the removal of forms by 5–9 h in terms of the early strength.
(5) The construction productivity was analyzed based on the elapsed time on concretes. The results were as follows. C24_E50_G20 was 68.24 h/cycle, which was superior to other concretes in terms of construction productivity. If C24_E50_G20 was applied to the structure works in a construction project, the construction period would be decreased compared to other concretes. Moreover, it was found that when C35_O100 was replaced with C24_G20 or C24_E50_G20, the economic and environmental impacts could be reduced by up to 15.16% and 38.00%, respectively.

The limitations of this study are as follows. First of all, it lacks a review of the mix proportions according to various EPC and GGBS replacement levels. Therefore, it is necessary to use a variety of materials for the economic review. In addition, there is a need to confirm the reproducibility of the early strength at the ready-mix concrete plant. Secondly, in terms of economic impact, C24_G20 was cheaper than C24_E50_G20; however, in terms of productivity for a 7-day cycle, it can be expected that C24_E50_G20 will be cheaper than C24_G20, so future research should be done to perform a productivity analysis between C24_G20 and C24_E50_G20 for a 7-day cycle.

The results of this study are expected to be used as basic data with reference to process management for the early removal of forms and the economical mix proportions of concrete at construction sites.

## Figures and Tables

**Table 1:** Physical properties of the used materials.

Material		Property
OPC		ASTM type I ordinary Portland cement Fineness: 330 m^2^/kg, density: 3150 kg/m^3^
EPC		ASTM type III early Portland cement Fineness: 488 m^2^/kg, density: 3160 kg/m^3^
GGBS		Ground granulated blast-furnace slag Fineness: 430 m^2^/kg density: 2860 kg/m^3^
Fine aggregate	S1	Sea sand, size: 5 mm Fineness modulus: 2.01, density: 2600 kg/m^3^, absorption: 0.79%
	S2	Crushed sand, size: 5 mm Fineness modulus: 3.29, density: 2570 kg/m^3^, absorption: 0.87%
Coarse aggregate		Crushed granitic aggregate Size: 25 mm, density: 2600 kg/m^3^, absorption: 0.76%
Chemical admixture	NP	Naphthalene superplasticizer, density: 1220 kg/m^3^
	PC	Polycarboxylic superplasticizer, density: 1260 kg/m^3^

**Table 2:** Chemical compositions on used binders.

Materials	Chemical Compositions (%)								L.O.I. ^(4)^
	CaO	Al_2_O_3_	SiO_2_	MgO	K_2_O	Fe_2_O_3_	SO_3_	Etc.	
OPC ^(1)^	60.34	4.85	19.82	3.83	1.08	3.3	2.9	0.86	3.02
EPC ^(2)^	61.44	4.72	20.33	2.95	0.95	3.42	3.73	0.79	1.67
GGBS ^(3)^	44.9	13	35.4	5.01	0.37	0.47	1.31	-	0.69

Note: OPC ^(1)^: ordinary Portland cement; EPC ^(2)^: early Portland cement; GGBS ^(3)^: ground granulated blast-furnace slag; and L.O.I. ^(4)^: loss on ignition.

**Table 3:** Experimental plan.

Type	Mix No.	W/B	Unit Weight of Binder (kg/m^3^)	Replacement of Binder (%)			Chemical Admixture	Evaluation Item
				OPC	EPC	GGBS		
I. Raw material	-	-	-	-	-	-	-	▪ Particle size distribution (%) ▪ Scanning electron microscope ▪ X-ray fluorescence ▪ X-ray diffraction ▪ Grading distribution (%)
II. Concrete	C35_O100 ^(1)^ C24_G20 C24_E50_G20	0.42 0.48 0.48	C35/444 ^(2)^ C24/340 C24/340	100 80 30	50	20 20	NP PC PC	▪ Compressive strength (MPa) - Cylinder mold (Ø100 × 200) - 1, 3, 7, 14, 28, 56, 91 day ▪ Chloride penetration depth (mm) ▪ Chloride ion diffusion coefficient (m^2^/s) ▪ Pore structure

Note: C35_O100 ^(1)^: Characteristic value of concrete 35 MPa, ordinary Portland cement 100% of binder; and C35/444 ^(2)^: characteristic value of concrete 35 MPa/unit weight of binder 444 kg/m^3^.

**Table 4:** Mixing proportions of concrete.

Mix ID.	W/B ^(1)^	S/a ^(2)^ (%)	Unit Weight (kg/m^3^)						NP (B × wt.%)	PC (B × wt.%)
			W ^(3)^	C ^(4)^	EPC	GGBS	S ^(5)^	G ^(6)^		
C35_O100	0.42	48.5	185	444	-	-	788	842	0.7	-
C24_G20	0.48	48.5	163	272	-	68	854	913	-	0.7
C24_E50_G20	0.48	48.5	163	102	170	68	854	913	-	0.7

Note: ^(1)^ W/B: Water to Binder; ^(2)^ S/a: Sand/aggregates; ^(3)^ W: Water; ^(4)^ Cement; ^(5)^ Sea sand + crushed sand; and ^(6)^ G: Gravel.

**Table 5:** Test methods for raw materials of binders.

Items	Materials	Evaluation Items	Test Methods
Raw material analysis	OPC EPC GGBS	Particle size distribution (%)	ASTM C204
		Scanning electron microscope	ASTM C1723
		X-ray fluorescence	ASTM C114
		X-ray diffraction	ASTM C457

**Table 6:** Test methods for engineering and durability properties of concrete.

Series	Evaluation Item	Test Method
Durability properties analysis	Chloride ion diffusion coefficient (10^−12^ m^2^/s)	NT Build 492
	Chloride penetration depth (mm)	NT Build 443
	Porosity (%)	ASTM D4404
	Maturity (D∙h)	ASTM C1074

**Table 7:** Data of the material unit costs and CO_2_ emissions.

Class	Materials				
	W ^(1)^	C ^(2)^	EPC ^(3)^	S ^(4)^	G ^(5)^
Material unit cost (US $/kg)	8.11 × 10^−4^	5.76 × 10^−2^	7.30 × 10^−2^	8.91 × 10^−3^	8.11 × 10^−3^
CO_2_ emission (CO_2_-kg)	1.02 × 10^−4^	9.44 × 10^−1^	3.77 × 10^−2^	5.03 × 10^−4^	9.18 × 10^−4^

Note: W ^(1)^: Water; C ^(2)^: Cement; EPC ^(3)^: Early Portland cement; S ^(4)^: Sea sand + Crushed sand; and G ^(5)^: Gravel.

**Table 8:** Fresh properties of concrete.

Mix ID.	Slump (mm)		Air Contents (%)	
	Initial Time	After 60 min	Initial Time	After 60 min
C35_O100	185	150	4.6	4.2
C24_G20	180	165	4.7	4.3
C24_E50_G20	180	165	4.6	4.5

## Data Availability

The data presented in this study are available in the manuscript and in the supplementary material and can be requested from the corresponding authors.

## Supplementary Materials

The following are available online at https://www.mdpi.com/article/10.3390/ma14174903/s1, Figure S1: The discrete event simulation diagram for analysis of the construction productivity, Table S1: The data of the crew members of structure works for the discrete event simulation, Table S2: The data of the working hours of structure works for the discrete event simulation.
