# Supplementary material for: Improving Marine Concrete Performance Based on Multiple Criteria Using Early Portland Cement and Chemical Superplasticizer Admixture
Source: Materials (Basel). 2021 Aug 28;14(17):4903. doi: 10.3390/ma14174903 (PMC8432681; doi:10.3390/ma14174903)
Supplement: Supplementary file 1 [file materials-14-04903-s001.zip › materials-1328381-supplementary.pdf]

materials

Supplementary materials

Improving Marine Concrete Performance Based on Multiple Criteria Using Early Portland Cement and Chemical Superplasticizer Admixture

Taegyu Lee [1,†], Jaehyun Lee [2,†], Jaewook Jeong [2,*] and Jaemin Jeong [2]

[1] Department of Fire and Disaster Prevention, Semyung University, 65, Semyeong-ro, Jecheon-si 27136, Korea; ltg777@semyung.ac.kr
[2] Department of Safety Engineering, Seoul National University of Science and Technology, 232 Gongneung-ro, Nowon-gu, Seoul 01811, Korea; archi0528@seoultech.ac.kr (J.L.); ss96011@seoultech.ac.kr (J.J.)
* Correspondence: jaewook.jeong@seoultech.ac.kr; Tel.: +82-2-970-6381
† These authors contributed equally to this work as first author.

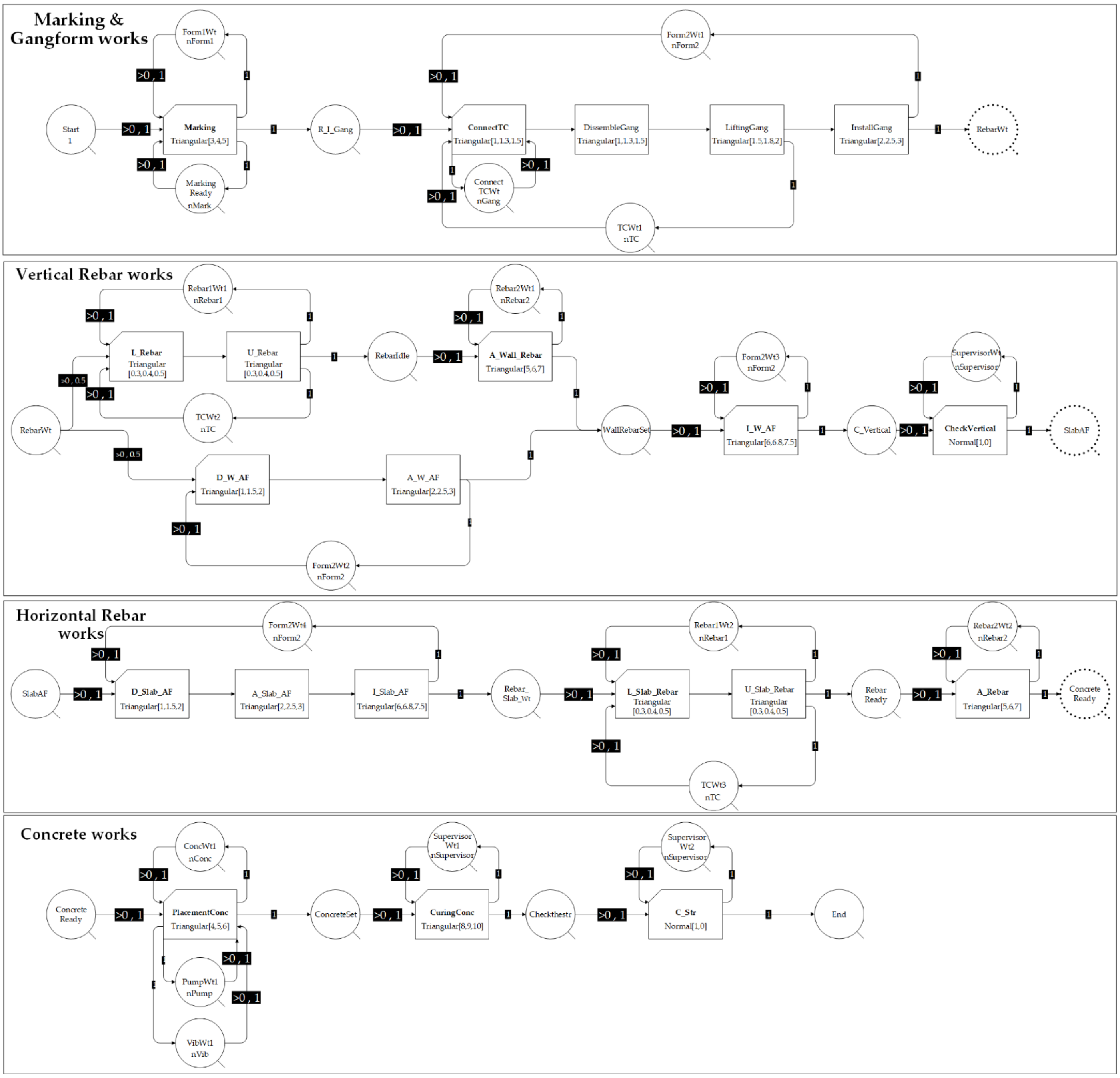

Figure. S1 The discrete event simulation diagram for analysis of the construction productivity

Table S1. The data of the crew members of structure works for the discrete event simulation.

Crew Member	Description	The Number of Workers
nMark	The number of Marking worker	1 (Unskilled Labour 1)
nForm1	The number of Formwork #1	2 (Foreman 1 + Form worker 1)
nForm2	The number of Formwork #2	8 (Foreman 1 + Form worker 7)
nTC	The number of Tower Crane	1 (Tower crane 1)
nRebar1	The number of Rebar worker #1	2 (Foreman 1 + Rebar worker 1)
nRebar2	The number of Rebar worker #2	5 (Foreman 1 + Rebar worker 4)
nSupervisor	The number of Supervisor	1 (Supervisor 1)
nConc	The number of Concrete worker	5 (Foreman 1 + Concrete worker 4)
nPump	The number of Pump car	1 (Pump car 1)
nVIb	The number of Vibrator	1 (Vibrator 1)

Table S2. The data of the working hours of structure works for the discrete event simulation.

Work Activity	Working Hours		
	Min	Mean	Max
Conducting marking	3	4	5
Connection with T/C	1	1.3	1.5
Disassemble gang form	1	1.3	1.5
Lifting gang form	1.5	1.8	2
Installation of gang form	2	2.5	3
Lifting rebar	0.3	0.4	0.5
Unloading rebar	0.3	0.4	0.5
Arranging rebar for wall	5	6	7
Disassemble wall al-form	1	1.5	2
Arranging wall al-form	2	2.5	3
Installation of wall al-form	6	6.8	7.5
Disassemble slab al-form	1	1.5	2
Arranging slab al-form	2	2.5	3
Installation of slab al-form	6	6.8	7.5
Arranging rebar for slab	5	6	7
Placement of concrete	4	5	6
Concrete curing and finishing	Based on the elapsed time		
Checking concrete strength	1	1	1
